# Early overnutrition reduces Pdx1 expression and induces β cell failure in Swiss Webster mice

**DOI:** 10.1038/s41598-019-39177-3

**Published:** 2019-03-06

**Authors:** Maria M. Glavas, Queenie Hui, Eva Tudurí, Suheda Erener, Naomi L. Kasteel, James D. Johnson, Timothy J. Kieffer

**Affiliations:** 1 Department of Cellular and Physiological Sciences, University of British Columbia, Vancouver, BC Canada; 2 Department of Surgery, University of British Columbia, Vancouver, BC Canada; 3 Present Address: Centro de Investigación Biomédica en Red de Diabetes y , Enfermedades Metabólicas Asociadas (CIBERDEM), Madrid, Spain

## Abstract

Childhood obesity and early rapid growth increase the risk for type 2 diabetes. Such early overnutrition can be modeled in mice by reducing litter size. We investigated the effects of early overnutrition and increased dietary fat intake on β cell function in Swiss Webster mice. On a moderate-fat diet, early overnutrition accelerated weight gain and induced hyperinsulinemia in pups. Early overnutrition males exhibited higher β cell mass but reduced islet insulin content and *Pdx1* expression. Males had a high diabetes incidence that was increased by early overnutrition, characterized by a progressive increase in insulin secretion as well as β cell death, indicated by histological analysis and increased circulating miR-375 levels. Females maintained normoglycemia throughout life. High-fat diet (HFD) increased diabetes incidence in males, whereas low-fat diet was completely protective. This protective effect was abolished in early overnutrition males transiently exposed to HFD in early life. Although Swiss Webster mice are not known to be diabetes-prone, the high diabetes incidence suggests an underlying genetic susceptibility that can be induced by overnutrition and increased dietary fat intake in early life. Thus, the nutritional environment in early life may impact long-term β cell function and increase diabetes risk, particularly in genetically susceptible individuals.

## Introduction

A major risk factor for obesity and type 2 diabetes is prolonged overnutrition, particularly with an obesogenic diet. In addition, evidence from epidemiological and animal studies suggests that an adverse *in utero* and early postnatal environment can increase the risk of type 2 diabetes^1–8^. Maternal obesity, high energy diet, and hyperglycemia during pregnancy have all been implicated in increased offspring diabetes risk^9,10^. Although it is difficult to investigate postnatal risk factors in humans without the confounding effects of *in utero* environment differences, both human and animal studies have noted increased diabetes risk with increased infant adiposity, early rapid growth, accelerated catch-up growth following intrauterine growth restriction (IUGR), and childhood obesity^2,11–13^. These observations suggest that the early developmental environment can have long-term effects on glucose homeostasis and ultimately contribute to disease. However, the effects of early overnutrition alone on the development of β cells and glucose homeostasis are unclear.

In humans, β cell mass rapidly increases after birth, with the greatest rate of expansion occurring before 2–5 years of age and the highest rate of β cell replication up to 2 months of age^14^. A similar pattern of postnatal expansion is observed in mice, with the highest rate of β cell mass expansion between birth and weaning, and peak β cell replication at 9 days of age^15^. It has been speculated that this early β cell mass expansion during infancy and childhood prepares the individual to cope with the metabolic demands of puberty and adulthood^14^. Consequently, insults that impair postnatal β cell mass expansion or functional development may reduce the capacity to meet later metabolic demands, leading to diabetes.

We investigated the effects of early overnutrition on β cells by employing a mouse model of early overnutrition, induced by reducing litter size, which reduces competition for maternal milk. Numerous studies have demonstrated that in rodents, this model results in accelerated body weight gain and adiposity in pups, with some studies showing alterations in glucose homeostasis, although phenotype varies by species and strain^16–22^. However, the effects of early overnutrition and dietary fat in early life on β cell development have not been thoroughly examined. In the Swiss Webster mouse, reduced litter size has previously been shown to increase susceptibility to HFD-induced obesity and insulin resistance^20^. We hypothesized that overnutrition and increased dietary fat intake during the early postnatal period in Swiss Webster mice would adversely affect β cell development and long-term function.

## Results

### Early Overnutrition in Pups Alters Body Composition and β Cell Mass

**Figure 1:**
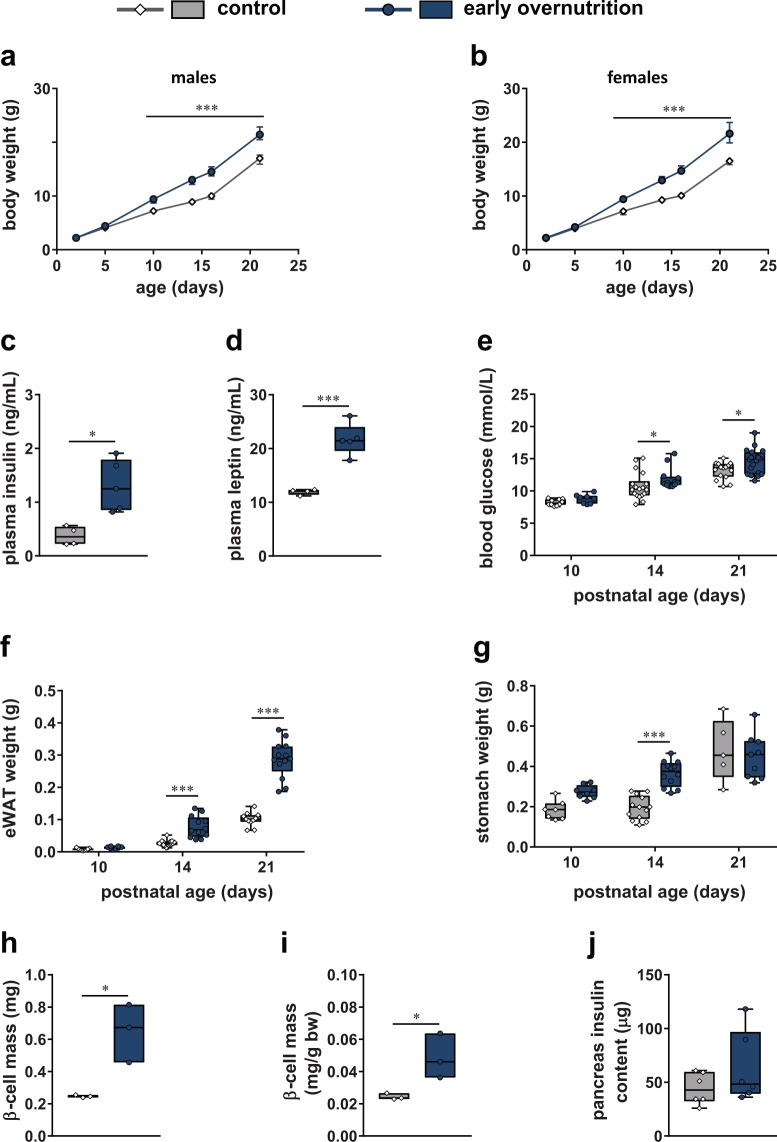
Effects of early overnutrition in pups. Body weight in male (**a**) and female (**b**) pups from postnatal day (P) 2 until weaning at P21 (n = 18–19 for males, n = 7–8 for females). Non-fasted plasma insulin (**c**) and plasma leptin (**d**) in P11 males. Non-fasted blood glucose (**e**), epididymal white adipose tissue (eWAT) weight (**f**), and full stomach weight (**g**) in P10, 14, and 21 male pups. β cell mass (**h**) and β cell mass normalized to body weight (**i**) in P14 male pups. Pancreatic insulin content (**j**) in P21 male pups. Data in (**a**) and (**b**) represent median ± interquartile range. Box and whisker plots (**c**–**g**, **j**) represent the interquartile range (box) and minimum to maximum values (whiskers), with line at the median. Floating bars (**h**,**i**) represent the range of values with line at the median. Analyses by two-way repeated measures ANOVA with Bonferroni post-hoc (**a**,**b**), two-way ANOVA with Bonferroni post-hoc (**e**–**g**), or unpaired t-test (**c**,**d**,**h**–**j**); *p < 0.05, ***p < 0.0005.

In both male and female pups (Fig. 1a,b), early overnutrition resulted in increased body weight gain such that early overnutrition pups weighed significantly more than control pups beginning at postnatal day (P) 10 through weaning at P21. Early overnutrition male pups exhibited elevated non-fasted insulin (Fig. 1c) and leptin (Fig. 1d) levels at P11, as well as increased non-fasted blood glucose levels at P14 and P21 (Fig. 1e). In addition, early overnutrition male pups had increased adiposity at P14 and P21, demonstrated by increased mass of the epididymal, retroperitoneal, and mesenteric fat pads, even when normalized to body weight (Fig. 1f, Table 1). Full stomach weight (Fig. 1g) was significantly increased at P14 in early overnutrition vs control male pups, reflecting their increased milk intake since the weight was mainly due to the stomach contents. Early overnutrition pups exhibited higher pancreas, liver, kidney, and spleen weight as well as body length at P14 (Table 1), suggesting a more rapid growth rate. β cell mass was significantly increased in early overnutrition male pups at P14 (Fig. 1h,i) even when normalized to body weight. However, total pancreas insulin content (Fig. 1j) measured at P21 did not differ between early overnutrition and control male pups.

**Table 1:** Tissue weights in postnatal day (P) 14 and P45 male mice.

Tissue/Measure	Control	Early Overnutrition	p-value
**P14**			
nasoanal length (cm)	6.6 (6.5–6.7)	7.0 (6.9–7.1)	**<0.001
eWAT (mg/g bw)	2.345 (2.090–3.033)	5.388 (3.703–8.159)	**<0.001
rpWAT (g)	0.014 (0.010–0.017)	0.033 (0.026–0.044)	**<0.001
rpWAT (mg/g bw)	1.379 (0.918–1.643)	2.580 (2.024–3.320)	**<0.001
mWAT (g)	0.045 (0.042–0.056)	0.074 (0.065–0.081)	**<0.001
mWAT (mg/g bw)	4.740 (4.080–5.170)	5.866 (5.014–6.152)	*0.011
pancreas (g)	0.022 (0.020–0.025)	0.029 (0.027–0.033)	*0.001
pancreas (mg/g bw)	2.110 (2.032–2.428)	2.220 (2.066–2.413)	0.707
liver (g)	0.399 (0.360–0.442)	0.557 (0.544–0.582)	**<0.001
liver (g/g bw)	0.038 (0.037–0.042)	0.043 (0.041–0.044)	*0.016
kidney (g)	0.068 (0.066–0.072)	0.088 (0.083–0.091)	**<0.001
kidney (mg/g bw)	6.541 (6.323–7.455)	6.584 (6.349–7.125)	0.689
spleen (g)	0.056 (0.049–0.063)	0.078 (0.065–0.104)	**<0.001
spleen (mg/g bw)	5.365 (4.714–6.108)	6.043 (5.138–7.998)	0.110
**P45**			
nasoanal length (cm)	10.3 (10.0–10.8)	10.5 (10.2–10.6)	0.796
eWAT (g)	0.559 (0.386–0.750)	1.019 (0.816–1.052)	*0.008
eWAT (mg/g bw)	14.66 (10.47–19.34)	22.03 (19.06–22.90)	*0.039
rpWAT (g)	0.184 (0.076–0.211)	0.206 (0.164–0.281)	0.199
mWAT (g)	0.372 (0.256–0.461)	0.598 (0.355–0.847)	0.091
pancreas (g)	0.364 (0.300–0.397)	0.395 (0.350–0.409)	0.272
liver (g)	1.693 (1.634–1.989)	2.101 (1.898–2.200)	0.061
kidney (g)	0.352 (0.308–0.387)	0.412 (0.394–0.423)	*0.014
kidney (mg/g bw)	9.395 (8.425–9.897)	9.258 (8.697–9.551)	0.908
spleen (g)	0.164 (0.122–0.181)	0.161 (0.131–0.172)	0.981

Values represent median (interquartile range), n = 11–13 per group at P14 and n = 5 per group at P45. P14 spleen (g and mg/g bw) and P14 and P45 kidney (mg/g bw) weights analyzed by Mann-Whitney, all others by unpaired t-test. eWAT: epididymal white adipose tissue; rpWAT: retroperitoneal white adipose tissue; mWAT: mesenteric white adipose tissue; bw: body weight. *p < 0.05, **p < 0.001.

### Glucose and Insulin Tolerance in Adults

**Figure 2:**
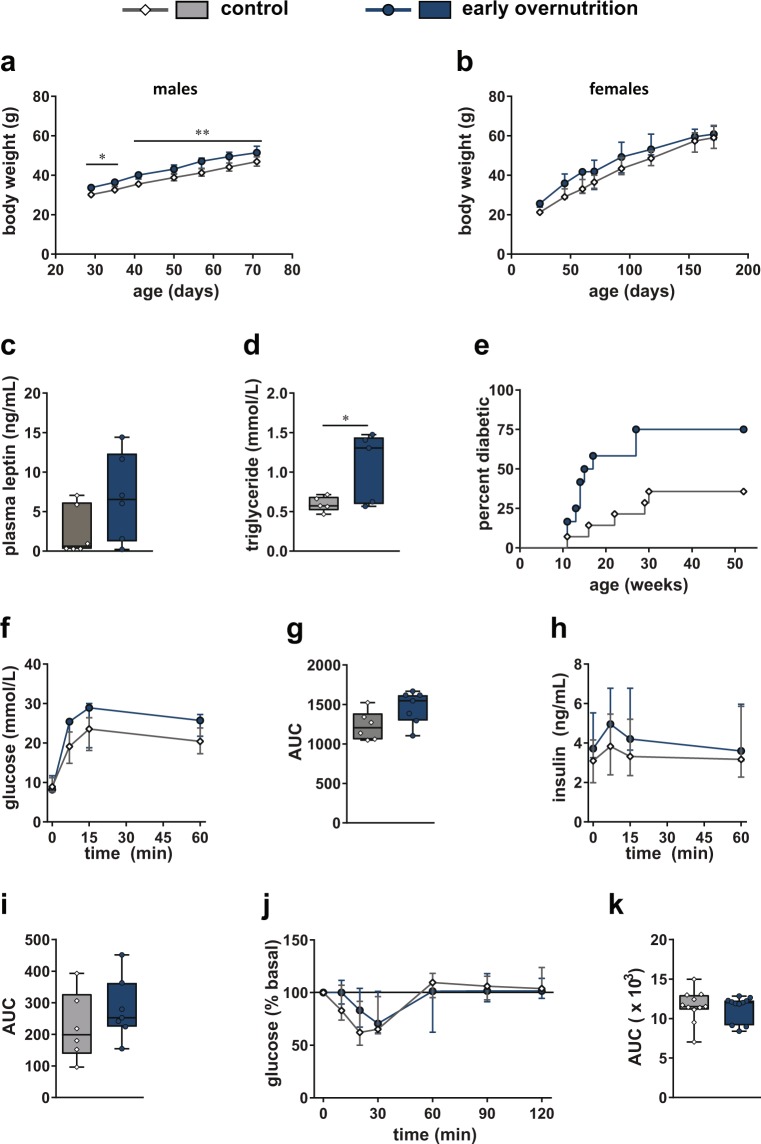
Glucose and insulin tolerance in adults. Body weight in males (**a**) to postnatal day (P) 71 (n = 15–16) and females (**b**) to P171 (in females, main effect of group with early overnutrition > control by two-way repeated measures ANOVA, p < 0.05; n = 7–8). Plasma leptin levels (**c**) and plasma triglyceride levels (**d**) at P50 in males, and diabetes incidence (**e**) in males to 52 weeks of age (p = 0.021 by log-rank test). Blood glucose response (**f**), glucose AUC (**g**), plasma insulin response (**h**), and insulin AUC (**i**) after intraperitoneal injection of 2 g/kg of glucose in 6 hr fasted male mice at P58. Blood glucose response normalized to baseline (**j**) and glucose AUC (**k**) in response to intraperitoneal injection of 0.75 U/kg insulin (Novolin) in 4 hr fasted male mice at P85. Data in (**a**,**b**,**f,h** and **j**) represent median ± interquartile range. Box and whisker plots (**c**,**d**,**g**,**i**,**k**) represent the interquartile range (box) and minimum to maximum values (whiskers), with line at the median. AUC: area under the curve with baseline = 0. Analyses by two-way repeated measures ANOVA with Bonferroni post-hoc (**a**,**b**) or unpaired t-test (**c**); *p < 0.05, **p < 0.005.

Both early overnutrition males and females maintained a small but significant increase in body weight, relative to their control counterparts, post-weaning and into adulthood (Fig. 2a,b). However, food intake did not differ (median (interquartile range; IQR) of daily caloric intake in control: 0.55 (0.52–0.65) kcal/g body weight, n = 7; early overnutrition: 0.53 (0.50–0.59) kcal/g body weight, n = 8; p = 0.141 by unpaired t-test, measured in males only). Plasma leptin did not differ in males at P60 (Fig. 2c). However, early overnutrition males had elevated plasma triglyceride levels at P50 (Fig. 2d) and a higher incidence of diabetes (Fig. 2e) compared to control males, with earliest onset of diabetes at 11 weeks of age, characterized by hyperglycemia, polyuria, and polydipsia. In contrast, females did not develop diabetes and maintained normal fasting glucose levels to P171 (median (IQR) in control: 6.8 (6.3–7.6) mmol/L; early overnutrition: 7.2 (6.5–7.7) mmol/L, p = 0.48 by unpaired t-test). Intraperitoneal glucose tolerance tests performed at P58, when no mice were diabetic, demonstrated no statistically significant differences in glucose or insulin responses between early overnutrition and control males (Fig. 2f–i). Although there was a trend toward increased glucose responses at this age, oral glucose tolerance tests in a separate cohort of mice showed no differences in glucose responses at P44 or P77 (Supplementary Fig. S1). In addition, early overnutrition and control males showed similar glucose responses during insulin tolerance tests at P85 (Fig. 2j,k), suggesting comparable degrees of insulin sensitivity.

### Islets in Non-Diabetic Adult Mice

**Figure 3:**
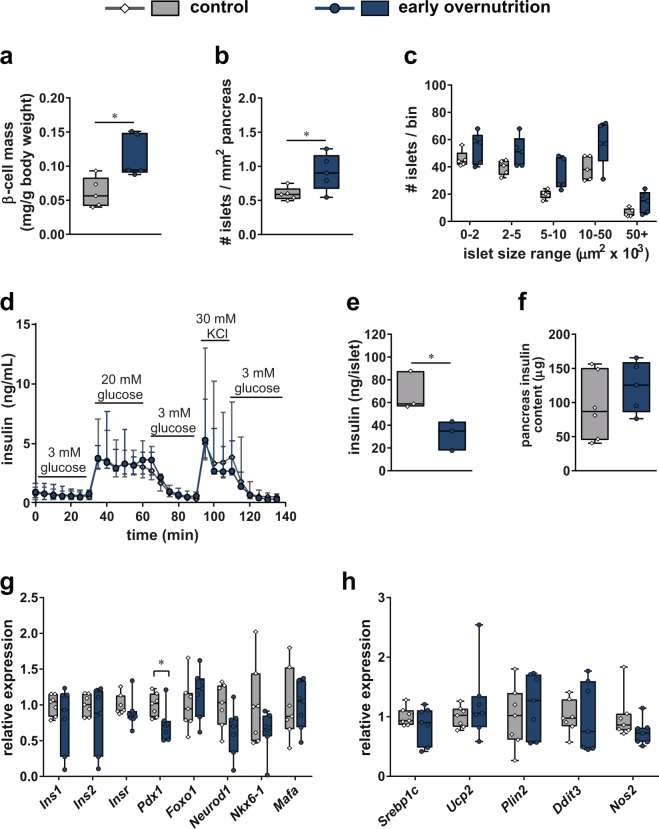
Islets in non-diabetic adult mice. β cell mass normalized to body weight (**a**), number of islets normalized to pancreas area (**b**), and islet size distribution (**c**) measured by insulin-positive area per islet, in non-diabetic early overnutrition vs control males at postnatal day (P) 100. Insulin secretion (**d**) of 80 size-matched isolated islets per mouse perifused with glucose (3 and 20 mM) and KCl (30 mM) at P80. Insulin content per islet (e**)** averaged from 5 size-matched islets per mouse at P94, and total pancreas insulin content (**f**) at P88. Expression of genes important for β cell function (**g**) and genes implicated in β cell lipotoxicity (*Srebp1c*, *Ucp2*, *Plin2*), endoplasmic reticulum stress (*Ddit3*, *Plin2*), and oxidative stress (*Nos2*) (**h**) in islets isolated at P50. Box and whisker plots (**a**–**c**,**f**-**h**) represent the interquartile range (box) and minimum to maximum values (whiskers), with line at the median. Line graph (**d**) represents median ± interquartile range. Floating bars (**e**) represent the range of values with line at the median. Analyses by unpaired t-tests (for **a**,**b**,**e**), or unpaired t-test with Bonferroni correction for multiple comparisons (**g**); *p < 0.05.

To investigate the effects of early overnutrition on β cell mass in adults, we examined insulin stained pancreas sections in non-diabetic males. Early overnutrition males exhibited significantly increased β cell mass at P100 compared to control males, even when normalized to body weight (Fig. 3a). This was due to an increase in the number of islets (Fig. 3b), whereas islet size distribution did not differ between early overnutrition and control (Fig. 3c). Isolated islets from non-diabetic mice showed similar responses to basal (3 mM) and high (20 mM) glucose levels and in response to 30 mM KCl depolarization (Fig. 3d). However, early overnutrition islets exhibited less insulin per islet (Fig. 3e). Total pancreas insulin content did not differ between early overnutrition and control mice (Fig. 3f), likely due to the reduced insulin content per islet despite the larger islet number in early overnutrition mice. To identify early alterations in β cells that may contribute to progressive dysfunction, we examined gene expression in islets from young (P50) non-diabetic mice. We observed no differences in islet mRNA expression of insulin 1 (*Ins1*), insulin 2 (*Ins2*), or insulin receptor (*Insr*) between early overnutrition and control males (Fig. 3g). However, gene expression of the key β cell transcription factor *Pdx1* was significantly decreased in early overnutrition vs control islets, with no difference in the β cell-enriched transcription factors *Foxo1*, *Nkx6-1*, and *Mafa*. *Neurod1* was marginally reduced in early overnutrition mice but did not reach a statistically significant difference (p = 0.0549). Genes that have been shown to be important in β cell lipotoxicity, endoplasmic reticulum (ER) stress, and oxidative stress (*Srebp1c*, *Ucp2*, *Plin2*, *Ddit3*, and *Nos2*)^23–27^ did not differ between early overnutrition and control islets (Fig. 3h).

### Early Overnutrition and β cell Death in Male Mice

**Figure 4:**
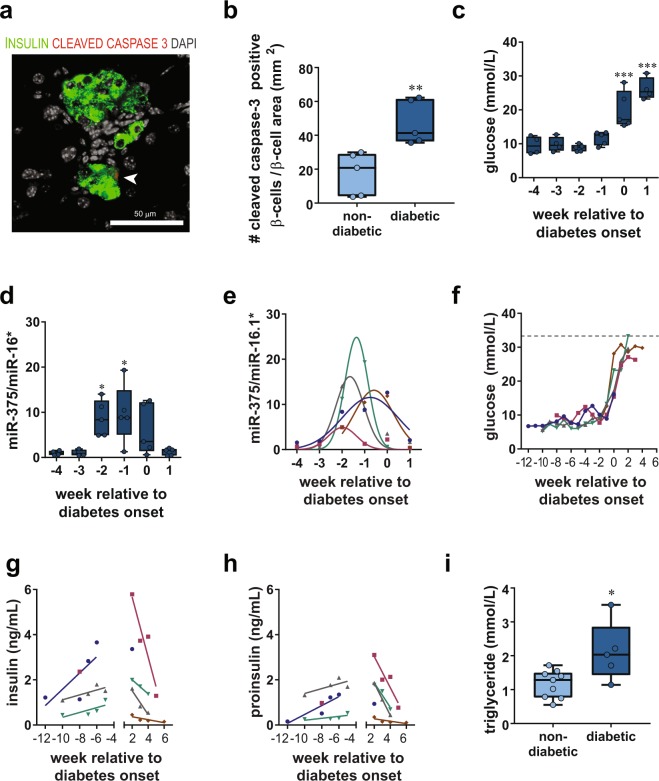
β cell death in male Swiss Webster mice. Cytoplasmic cleaved caspase 3 staining (**a**, red) was rarely observed in insulin (green) positive cells in young (5 week) non-diabetic early overnutrition males but levels were increased in recently diabetic early overnutrition males (14 week; double-labeled insulin and cleaved caspase-3 cell indicated by white arrow). This was quantified (**b**) and normalized to β cell area, determined as the area in mm^2^ of positive insulin immunoreactivity per section (**p < 0.01 by unpaired t-test). Diabetes induced by early overnutrition resulted in elevated blood glucose (**c**) at 0 and 1 week compared to −4 weeks relative to diabetes onset (***p < 0.001 by one-way ANOVA). Levels of miR-375 (**d**) in plasma (normalized to miR-16*) were increased at −2 and −1 week compared to −4 weeks relative to diabetes onset (*p < 0.05 by one-way ANOVA). Gaussian distribution fit (**e**) of miR-375 plasma levels (normalized to miR-16*) in individual mice showing peak miR-375 levels ranging from −2 to 0 weeks relative to diabetes onset (unpaired t-test of mean amplitude relative to 0: p < 0.05). Fasting (4 hr) blood glucose (**f**), plasma insulin (**g**), and plasma proinsulin (**h**) levels in individual mice before and after diabetes onset. Both insulin and proinsulin increased prior to diabetes onset (slopes > 0 by one-tailed t-tests, p < 0.05) and decreased after diabetes onset (slopes < 0 by one-tailed t-tests, p < 0.05). Diabetes resulted in increased plasma triglyceride levels (**i**) in recently diabetic relative to non-diabetic 14 week old early overnutrition males. Box and whisker plots (**b**,**c**,**d**,**i**) represent the interquartile range (box) and minimum to maximum values (whiskers), with line at the median. Data in panels c through h are from 5 early overnutrition male mice that developed diabetes, with color assignment per mouse matched for panels e through h. Scale bar = 50 µm.

To determine whether diabetes in early overnutrition mice was due to increased apoptosis of β cells, we quantified insulin immunoreactive cells that colocalized with cleaved caspase-3, a specific marker for cells undergoing apoptosis^28^. Diabetes induced an increase in cleaved caspase-3-positive β cells (Fig. 4a,b), suggesting that β cell death by apoptosis contributed to diabetes progression. To better assess the dynamics of β cell death relative to disease onset, we measured circulating miR-375, a marker of β cell death^29,30^, once a week and compared it to glucose levels. In early overnutrition male mice, fasting glucose was stable in the weeks prior to diabetes (Fig. 4c,f), yet miR-375 was increased in the 2 weeks prior to diabetes onset (Fig. 4d,e). The peak of circulating miR-375 levels (Fig. 4e) varied among individual mice from −2 to 0 weeks relative to diabetes onset (age of onset ranged from P51 to P116), and levels dropped in the week following diabetes onset. To further assess the status of β cells in relation to diabetes onset, we measured both insulin and proinsulin levels in the same mice prior and following diabetes onset (Fig. 4g,h). Due to the large plasma volume required for measuring miR-375 levels, there was not sufficient plasma to measure these hormones at the same timepoints as miR-375 (i.e., −4 to 1 week relative to diabetes onset). Nonetheless, we observed that in individual mice both insulin and proinsulin rose prior to diabetes onset, suggesting progressive β cell dysfunction, and declined following diabetes onset, likely as a consequence of the β cell death indicated by a rise in circulating miR-375 levels. We did not observe a change in the proinsulin/insulin ratio over time (data not shown), suggesting that processing of proinsulin to insulin remained stable. Plasma triglyceride levels were stable in the weeks prior to diabetes onset (data not shown) but were significantly elevated in diabetic mice as expected (Fig. 4i).

### Early Overnutrition and Islet Morphology

**Figure 5:**
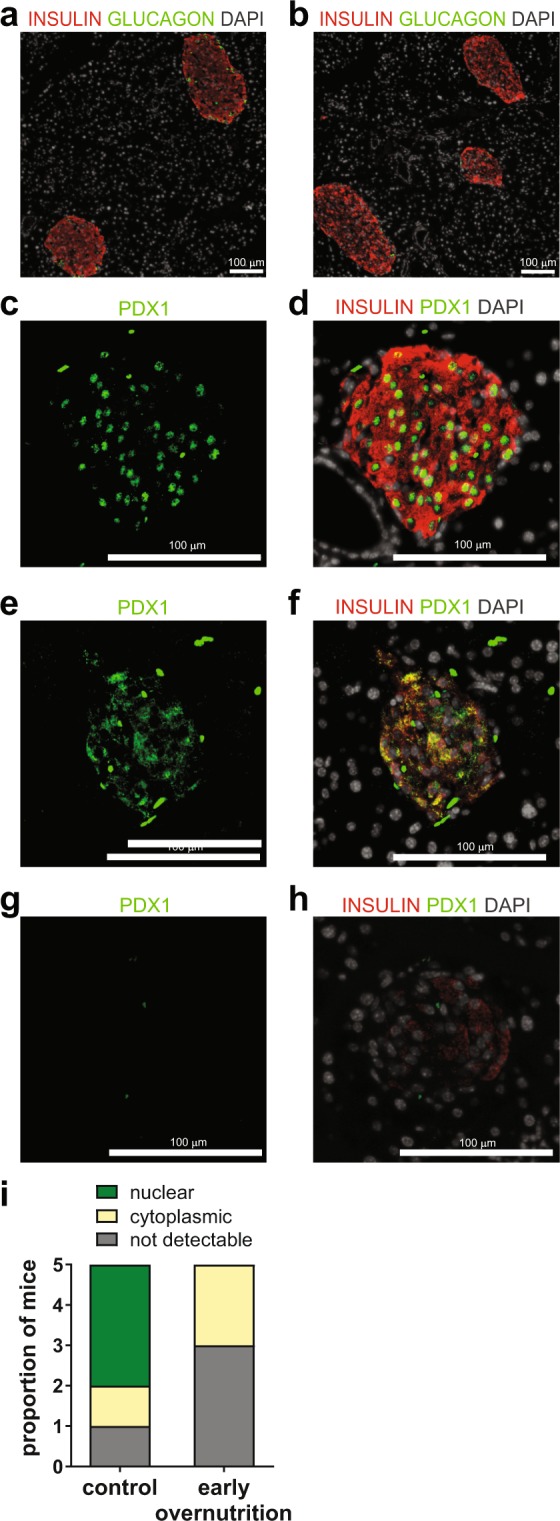
Islet morphology and Pdx1 in non-diabetic mice. Insulin (red) and glucagon (green) staining in non-diabetic control (**a**) and non-diabetic early overnutrition (**b**) mice at P100, demonstrating similar islet morphology (white: DAPI). Pdx1 staining (green) in a P100 non-diabetic control mouse (**c)** and Pdx1 (green) merged with insulin (red) (**d**) showing predominantly nuclear localization of Pdx1. P100 non-diabetic early overnutrition mice showing (**e**,**f**) mainly cytoplasmic staining of Pdx1 (green) colocalized with insulin in red, or non-detectable Pdx1 (**g**) and colocalization with insulin in red (**h**). All early overnutrition mice exhibited either cytoplasmic or non-detectable Pdx1 immunoreactivity (**i**), quantified as the predominant Pdx1 localization observed in each of 5 mice per group. Scale bars = 100 µm.

**Figure 6:**
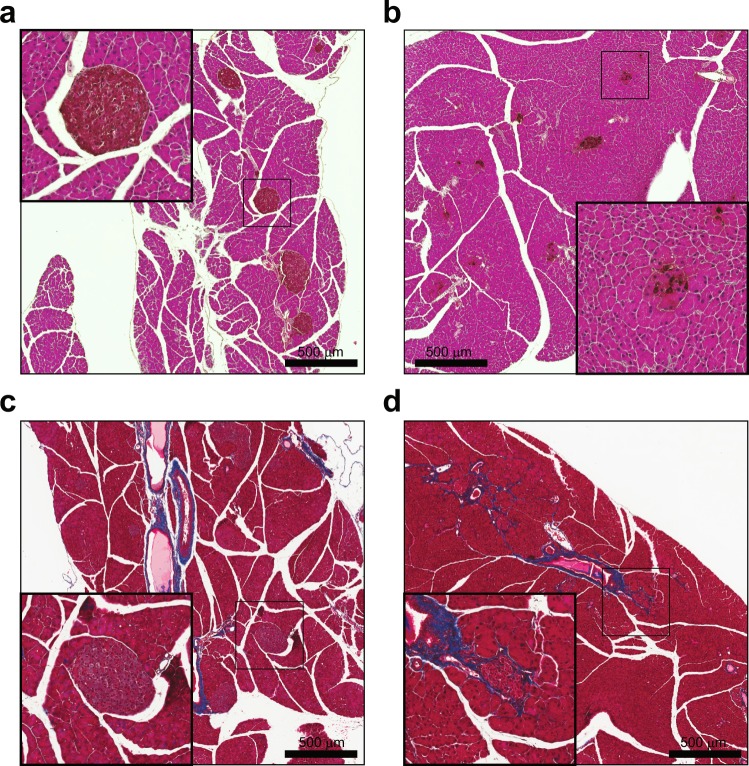
Islet morphology in diabetic mice. DAB-labelled insulin with an H&E counterstain demonstrated abundant insulin staining in a control non-diabetic mouse (**a**) and weaker and significant loss of insulin staining in a diabetic early overnutrition mouse (**b**). Masson’s trichrome stain in a non-diabetic control mouse (**c**) and a diabetic early overnutrition mouse (**d**) showing fibrosis, indicated by collagen staining in blue. All mice were 100 days of age. DAB: 3,3′-diaminobenzidine. Scale bars = 500 µm.

Islets from non-diabetic early overnutrition and non-diabetic control mice generally exhibited similar and normal morphology (Fig. 5a,b), although insulin immunoreactivity in early overnutrition islets tended to be more heterogeneous, with cell staining typically ranging from faint to intense within the same islet. To determine whether the observed reduction in *Pdx1* gene expression in early overnutrition mice translated to reduced Pdx1 content in β cells, we co-stained pancreas sections with insulin and Pdx1. Pdx1 staining had the expected nuclear localization (Fig. 5c,d,i) in 3 of 5 non-diabetic control mice, with cytoplasmic or not detectable staining in the other two. In contrast, non-diabetic early overnutrition mice did not exhibit nuclear Pdx1 staining and instead had either predominantly cytoplasmic (Fig. 5e,f,i) or undetectable staining (Fig. 5g,h,i). Insulin immunoreactivity was reduced in P100 diabetic early overnutrition and control mice (Fig. 6a,b) and there was notable fibrosis surrounding and within islets (Fig. 6c,d). We observed no signs of immune cell infiltration (insulitis, peri-insulitis) in the pancreas of non-diabetic, pre-diabetic, or diabetic mice by examination of H&E stained sections or by immunofluorescence, using CD45 as a general immune cell marker and CD3 as a T-cell specific marker (data not shown). This suggests that the loss of β cells in these mice is not due to autoimmune attack. Immunofluorescence staining for known markers of β cell dedifferentiation, Aldh1a3 and L-myc^31,32^, resulted in negligible immunoreactivity in islets of pre-diabetic and diabetic mice (data not shown), suggesting that the loss of insulin staining is not due to dramatic dedifferentiation of β cells.

### High-Fat Diet and Early Overnutrition

**Figure 7:**
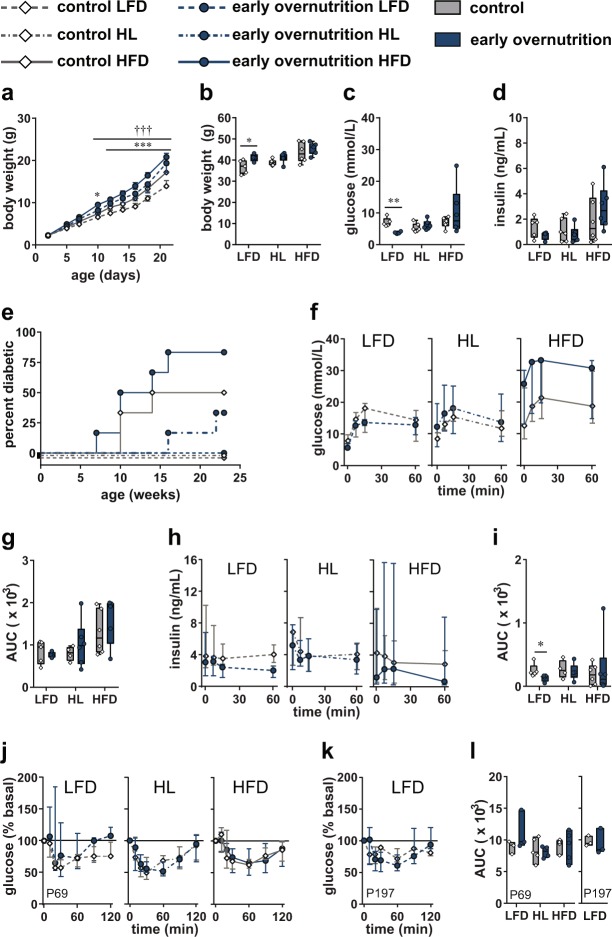
High-fat diet and early overnutrition. Body weight in male pups (**a**) maintained on LFD or HFD from postnatal day (2) to weaning at P21 (control LFD n = 5, early overnutrition LFD n = 4, control HFD n = 12, early overnutrition HFD n = 12). P50 body weight (**b**), fasting blood glucose (**c**), and fasting insulin (**d**) in control and early overnutrition males maintained on either LFD or HFD throughout life or switched from HFD to LFD at P21 (HL group). Diabetes incidence (**e**) to 23 weeks of age. Blood glucose response (**f**), glucose AUC (**g**), plasma insulin response (**h**), and insulin AUC (**i**) after gavage of 2 g/kg of glucose in 6 hr fasted mice at P121. Blood glucose response normalized to baseline (**j**) and glucose AUC (**l**, left side) in response to intraperitoneal injection of 0.75 U/kg insulin in 4 hr fasted mice at P69. Blood glucose response normalized to baseline (**k**) and glucose AUC (**l**, right side) in response to intraperitoneal injection of 0.75 U/kg insulin in 4 hr fasted control LFD and early overnutrition LFD mice at P197. Line graphs (**a**,**f**,**h**,**j**,**k**) represent the median ± interquartile range. Box and whisker plots (**b**,**c**,**d**,**g**,**i**) represent the interquartile range (box) and minimum to maximum values (whiskers), with line at the median. Floating bars (**l**) represent the range of values with line at the median. LFD: low fat (10%) diet; HFD: high fat (45%) diet beginning at P2; AUC: area under the curve with baseline = 0. *p < 0.05, **p < 0.005, ***p < 0.0005 for early overnutrition LFD vs control LFD by two-way repeated measures ANOVA (**a**) or unpaired t-test with Bonferroni correction for multiple comparisons (**b**,**c**,**i**); ^†††^p < 0.0005 for early overnutrition HFD vs control HFD by two-way repeated measures ANOVA (**a**).

To investigate whether dietary fat content influenced the effects of early overnutrition on β cell function, we compared early overnutrition and control males whose mothers were fed either LFD or HFD throughout lactation with offspring weaned onto the same diet (LFD and HFD groups). In addition, to investigate the effects of increased dietary fat that was limited to the preweaning period, a subset of mice weaned from dams on HFD were switched LFD for the remainder of their life (HL group). Early overnutrition male pups, from dams consuming either LFD or HFD during lactation, exhibited significantly increased body weight relative to their control male counterparts, beginning at P10 through weaning at P21 (Fig. 7a). At P50, early overnutrition LFD males maintained a higher body weight relative to control LFD mice; however, in mice switched from HFD to LFD at weaning (HL group), and in mice remaining on HFD, body weight no longer differed between early overnutrition and control mice (Fig. 7b). Fasting glucose (Fig. 7c) was significantly lower in early overnutrition LFD vs control LFD mice with no difference in HL or HFD-fed mice. Fasting insulin levels (Fig. 7d) did not differ between early overnutrition and control mice within any dietary condition. HFD promoted the development of diabetes and this was exacerbated by early overnutrition, such that diabetes incidence was induced in a subset of early overnutrition HFD, control HFD, and early overnutrition HL mice, whereas LFD prevented diabetes in both early overnutrition and control (Fig. 7e). During an oral glucose tolerance test (Fig. 7f–i), glucose responses did not differ between early overnutrition and control mice, but insulin responses were lower in early overnutrition LFD relative to control LFD. Overall, insulin responses did not exhibit the expected significant rise in response to glucose, suggesting either general dysregulation of insulin secretion in all groups or selection of inappropriate sampling timepoints that did not capture the peak of glucose-stimulated insulin secretion. During insulin tolerance tests at P69 (Fig. 7j,l), glucose responses did not differ between early overnutrition and control mice. A second insulin tolerance test performed at P197 in LFD-fed mice only (Fig. 7k,l) showed that both control and early overnutrition mice maintained insulin sensitivity. HFD-fed mice could not be included at this age since many had been euthanized at an earlier age due to diabetes.

## Discussion

We have demonstrated that overnutrition and increased dietary fat in early life can promote the development of diabetes. In Swiss Webster male mice, this diabetes is characterized by reduced *Pdx1* expression and β cell dysfunction, in the absence of insulin resistance even in HFD-fed mice, that progresses to β cell death. In contrast, female mice maintained normoglycemia throughout life. Since litter size reduction in other rodent strains does not typically induce such profound β cell death, Swiss Webster mice likely have a genetic susceptibility to β cell dysfunction and diabetes. Our findings suggest that the nutritional environment during early life may impact long-term β cell function and increase the risk of diabetes, particularly in genetically susceptible individuals.

As previous studies have shown^17,20,22^, we observed accelerated weight gain in both early overnutrition males and females with reduced litter size, as well as hyperinsulinemia and hyperleptinemia, relative to their control counterparts. We also observed elevated β cell mass relative to controls in males, even when normalized to body weight. Slightly heavier body weight, higher adiposity, as well as disproportionally higher β cell mass persisted into adulthood in early overnutrition males. Despite higher β cell mass and more islets in adult early overnutrition compared to control males, total pancreas insulin content did not differ, likely due to the reduced insulin content per islet. Although isolated islets from early overnutrition males responded normally to a short-term exposure to high glucose and KCl, the low insulin reserves per islet are likely insufficient to respond to prolonged stimulants, such as chronic HFD, making them more susceptible to β cell failure.

We observed a high rate of overt diabetes in Swiss Webster males, with higher incidence in early overnutrition compared to control males. Diabetes was not observed in females, despite experiencing a similar acceleration in body growth in early life. A previous study also observed diabetes in male, but not female, Swiss Webster mice, in a colony selectively bred for diabetes susceptibility^33^. The reason for this sexual dimorphism is unclear; however, estrogens are known to have protective effects against the development of diabetes by increasing β cell insulin content^34^, increasing insulin sensitivity^35,36^, and protecting against β cell apoptosis^37^. Young, non-diabetic early overnutrition males exhibited increased plasma triglyceride levels, suggesting dysregulated lipid homeostasis. However, glucose tolerance and insulin tolerance did not differ significantly from control males. This suggests that the β cell dysfunction in these mice is not secondary to obesity-induced insulin resistance, as often occurs in mouse models in response to chronic HFD exposure.

The reduced *Pdx1* expression that was observed in young early overnutrition males and the later development of low islet insulin content, suggests a primary β cell dysfunction induced by early overnutrition. This occurred despite the rapid β cell mass expansion observed in early overnutrition pups. Interestingly, the decrease in *Pdx1* expression occurred prior to changes in insulin expression. *Pdx1* is a key transcription factor required for normal pancreas development^38–40^. In humans, mutations in the *PDX1* gene can lead to maturity onset diabetes of the young^41^, and reduced *Pdx1* levels in mice have been shown to increase β cell susceptibility to apoptosis and HFD-induced ER stress^38,42^. Previous studies found that IUGR results in progressive reduction in *Pdx1* levels due to epigenetic silencing, and the increased diabetes incidence in this model could be prevented by treatment in early life with exendin-4 that increases *Pdx1*^43,44^, suggesting that reduced *Pdx1* during development can induce diabetes. Thus, reduced *Pdx1* levels in early overnutrition mice could be an early contributor to β cell dysfunction and render them more susceptible to metabolic stressors. The marginally reduced *Neurod1* expression could also contribute to this dysfunction, since both Pdx1 and NeuroD1 act in concert to regulate insulin expression^45^. Predominantly cytoplasmic localization of Pdx1, as observed in some early overnutrition mice, has previously been observed in rats exposed to high glucose and lipids^46^, and after 8 weeks of consuming 58% HFD in C57BL/6J mice^47^. Reduced *Pdx1* expression and immunoreactivity were observed at an age when early overnutrition mice did not exhibit differential glucose levels relative to control. Since elevated plasma triglycerides were observed in young early overnutrition males, it is possible that elevated lipid, but not glucose, levels may have contributed to the observed cytoplasmic Pdx1 localization. Although we did not detect increased expression of genes implicated in β cell lipotoxicity in young, non-diabetic early overnutrition mice, it may be that lipotoxic effects occurred at a later age.

The rise in plasma miR-375 levels observed in Swiss Webster mice prior to diabetes onset is indicative of β cell death, since dying β cells release miR-375 into the circulation^29^. The increased cleaved caspase-3 in β cells of diabetic mice suggests that cell death is at least in part due to apoptosis. In addition, the presence of fibrosis in and around islets is likely a consequence of this cell loss. Since mice developed diabetes at different ages, to better understand the progression of diabetes we tracked individual mice relative to diabetes onset. This revealed a progressive increase in insulin and proinsulin levels leading up to diabetes onset, suggestive of worsening β cell function. A progressive decrease in insulin and proinsulin occurred following diabetes onset and was therefore associated with hyperglycemia and increased β cell death. The susceptibility to overnutrition-induced β cell death in Swiss Webster mice makes this strain a useful model to study progression to diabetes, relative to the more commonly used C57BL/6 mice that exhibit relatively low rates of measurable β cell death on HFD despite developing obesity, hyperglycemia, and insulin resistance^48,49^. Indeed, a low dose of the β cell toxin streptozotocin is often combined with HFD in C57BL/6 mice to better mimic the β cell death that occurs in humans with type 2 diabetes^50^.

To investigate the role of dietary fat intake on diabetes susceptibility and its interaction with early overnutrition, control and early overnutrition offspring were raised either on HFD or LFD. HFD-feeding induced diabetes in both control and early overnutrition mice, whereas LFD was completely protective. In fact, early overnutrition mice maintained on LFD throughout life had improved glucose tolerance despite having a heavier body weight than control LFD mice, suggesting that early overnutrition could even be beneficial as long as dietary fat is kept low. HFD exposure only during the preweaning period prevented these beneficial effects of early overnutrition, suggesting that the preweaning period is a critical time of susceptibility to early overnutrition and dietary fat-induced β cell dysfunction. Whereas total fat content of milk remains high regardless of the mother’s level of dietary fat intake, the composition of fatty acids in milk varies to reflect that in the diet^51^. The HFD we employed had a higher proportion of saturated (mainly palmitic and stearic acids) and monounsaturated (mainly oleic acid) fatty acids compared to the LFD. Previous studies have observed that β cells are particularly sensitive to prolonged elevations in saturated fatty acids, with chronic palmitate exposure resulting in impaired β cell function including decreased *Pdx1* expression and increased ER stress^52–54^. Our studies suggest that increased exposure to saturated and/or monounsaturated fatty acids specifically during the early postnatal period may have long-term detrimental effects on β cell function.

In a previous study, reduced litter size in Swiss Webster males did not result in overt diabetes and β cell death, and instead mice were more prone to HFD-induced obesity and insulin resistance^20^. However, these mice were of different origin from a different vendor (Simonsen Laboratories Gilroy, CA), so may not have the same genetic susceptibility or microbiome composition as the mice in the present study. Alternatively, since HFD in the previous study began at 6 weeks of age instead of shortly after birth, moderate to high dietary fat intake during the early postnatal period, as in the present study, is required to see the detrimental effects on β cell function that lead ultimately to β cell death in the absence of insulin resistance. This is supported by the increased diabetes incidence observed in early overnutrition offspring exposed to HFD only during the preweaning period.

Swiss Webster mice appear to have a genetic susceptibility that predisposes them to diabetes, induced by metabolic challenges such as overnutrition and increased dietary fat in early life, during the period of rapid β cell mass expansion. Despite an early increase in β cell mass induced by early overnutrition, the β cell dysfunction reflected in the reduced islet insulin content and reduced *Pdx1* expression eventually leads to β cell death. Our findings may offer insight into potential detrimental effects that could occur in humans, particularly those with a genetic susceptibility for primary β cell dysfunction, resulting in adverse consequences from overnutrition in infancy and/or childhood. A better understanding of the underlying mechanisms mediating the development of β cell dysfunction in the Swiss Webster mouse, and the time frame in which they occur, may help us identify at-risk children that show a similar susceptibility, allowing for early interventions aimed at preventing long-term adverse outcomes.

## Methods

### Animals and Tissue Harvest

Mice were housed under a 12-hr light, 12-hr dark cycle (lights on at 0700 h), with constant temperature and humidity and *ad libitum* access to food and water. Pregnant (16–17d) Swiss Webster mice (Model #SW-F) were purchased from Taconic (Hudson, NY) and maintained on a moderate fat (26.1 kcal% fat) standard chow diet (5LJ5 diet, Lab Diet, St. Louis, MO). Two days post-delivery, litters were culled to 10 pups for the control group. For the early overnutrition group, litters were initially culled to 6 pups at P2, and then further culled to 3 pups at P5. This stepwise reduction in litter size reduces the chances of insufficient maternal milk production. Groups were assigned such that pup body weights did not differ between control and early overnutrition groups at P2. Pups were weaned onto the same diet as the mother (5LJ5). For the HFD study, pregnant Swiss Webster mice purchased from Taconic were placed on 10 kcal% low-fat diet (LFD; D12450B, Research Diets, Inc, New Brunswick, NJ). Two days post-delivery, dams were either maintained on LFD or switched to 45 kcal% lard-based HFD (D12451, Research Diets, Inc.), with litters culled as above. All offspring were weaned at P21, with mice from the LFD and HFD groups maintained on the same diet as their mother throughout life. To examine long-term effects of HFD that was limited to the preweaning period, a subset of mice weaned from HFD-fed dams was switched to LFD at P21 (HL group).

Since only male Swiss Webster mice developed diabetes, detailed metabolic assessments in adults were performed only in male mice. Adult tissue collection was performed after a 4 hr fast (0900 h to 1300 h) and all tissues were weighed immediately upon dissection. Pup tissue and trunk blood collection was performed in the AM in non-fasted male mice after isoflurane anesthesia. Tissues for immunohistochemistry and histology were post-fixed in 4% paraformaldehyde followed by paraffin-embedding and sectioning at 5 µm by Wax-It Histology Services Inc. (Vancouver, Canada). For total pancreas insulin content, whole pancreas was incubated in −20 °C 0.18 M HCl/70% ethanol, homogenized, then incubated again at −20 °C overnight. Following centrifugation, the aqueous solution was neutralized 1:2 with 1 M Tris, pH 7.5. All procedures with animals were approved by the University of British Columbia Animal Care Committee and carried out in accordance with the Canadian Council of Animal Care guidelines.

### Metabolic Assessments

Blood glucose values were determined with a OneTouch Ultra 2 glucometer (LifeScan, Inc., Burnaby, Canada) measured from saphenous vein blood. Fasting (4 hr) glucose and body weight were monitored weekly beginning at P30-35. Diabetes onset was defined as the first day of two consecutive fasting glucose measures ≥ 20 mM (or ≥ 15 mM for the miR-375 analysis study to match previous study)^29^. Food intake (averaged over 7 days) was measured in individually housed mice (aged P60 to 80) by weighing food hoppers daily. For glucose tolerance tests, 6 hr fasted mice were given 2 g/kg glucose either by intraperitoneal injection or oral gavage, and blood collected prior to glucose administration and at indicated times after injection into heparin-coated capillary tubes. For insulin tolerance tests, 4 hr fasted mice were injected intraperitoneally with 0.75 U/kg insulin (Novolin ge Toronto, Novo Nordisk Canada, Mississauga, Canada), with glucose measures conducted at 0, 10, 20, 30, 60, 90, and 120 min post-injection. For assessment of circulating miR-375 levels, ~100 µL of blood was collected from the saphenous vein into EDTA-coated capillary tubes. This was performed once per week, beginning at P32, in early overnutrition males maintained on 5LJ5 diet. Plasma was stored at −80 °C prior to RNA isolation.

### Islet Isolation

Islets were isolated as previously described^55^ using Type XI collagenase (1000 units/mL; #C7657, Sigma-Aldrich, St. Louis, MO) injected into the pancreatic duct. Following digestion and filtration, islets were picked with a pipette in three rounds to remove any remaining acinar tissue. For islet RNA isolation, ~100 islets per mouse were placed in a 1.5 mL tube and spun down at 200 x g for 1 min. Excess buffer was removed and then islets were homogenized in 600 µL RLT Buffer (Qiagen, Hilden, Germany) using a 1 mL syringe with a 21 gauge 1” needle. Tubes were then frozen on dry ice and stored at −80 °C for RNA isolation.

Islet perifusions were performed as previously described^38^. Briefly, 80 size-matched islets per mouse were perifused with a HEPES-buffered Krebs-Ringer buffer under temperature and CO_2_-controlled conditions. Perifusate was collected every 5 min under basal glucose (3 mM), high glucose (20 mM), and 30 mM KCl conditions, and insulin secretion analyzed by radioimmunoassay. For islet insulin content, 5 islets per mouse were acid-ethanol extracted as described above and stored at −20 °C.

### Pancreas Immunohistochemistry and Histology

All primary antibodies and dilutions used for immunohistochemistry are listed in Supplementary Table S1. For immunofluorescence staining, slides were deparaffinized and hydrated in xylene washes followed by graded ethanol washes. Slides were then washed in phosphate-buffered saline (PBS) followed by 10–15 min incubation in 10 mM sodium citrate/0.05% Tween 20, pH 6.0 at 95 °C. After cooling, slides were incubated for 10 min with DAKO Protein Block Serum-Free (Agilent Technologies, Inc, Santa Clara, CA) at room temperature and then overnight at 4 °C with primary antibody diluted in Dako Antibody Diluent (Agilent Technologies, Inc.). Following washes in PBS, slides were incubated in Alexa Fluor 488 or 594 secondary antibodies (Thermo Fisher Scientific, Waltham, MA) diluted in Dako Antibody Diluent and then washed in PBS. Slides were coverslipped with VECTASHIELD HardSet Mounting Medium with DAPI (Vector Laboratories, Burlingame, CA). Images were captured with the ImageXpress Micro High-Content Imaging system and MetaXpress 5.3.0.5 software (Molecular Devices, Sunnyvale, CA). For chromogenic staining of insulin, the procedure was as above on day 1 with the addition of a 12 min incubation in 3% H_2_O_2_ and 10 min PBS wash prior to the blocking step. On day 2, following washes in PBS, slides were incubated in biotinylated anti-guinea pig IgG (1:200, Jackson ImmunoResearch, West Grove, PA) diluted in Dako Antibody Diluent, for 1 hr at room temperature in a humid chamber. Slides were then washed in PBS, incubated for 30 min with R.T.U. VECTASTAIN ABC Reagent (Vector Laboratories), washed for 10 min in PBS, then incubated in 3,3′-diaminobenzidine (DAB) Peroxidase (HRP) Substrate solution (Vector Laboratories). Slides were counterstained with hematoxylin and eosin (H&E), dehydrated through a graded ethanol and xylene series, and then coverslipped with Vectamount Permanent Mounting Medium (Vector Laboratories).

β cell mass was analyzed in three pancreas sections per mouse, at least 200 µm apart, on insulin-DAB stained sections for P14 pups and immunofluorescent insulin-labeled sections in adults. Image threshold was set such that weak insulin staining within an islet was considered positive. Islet size was quantified as the insulin-positive area per islet. H&E staining and Masson’s trichrome staining on adult pancreas sections were performed by Wax-it Histology Services Inc. (Vancouver, Canada).

### RNA Isolation and quantitative RT-PCR

Total RNA was isolated from islets using the Qiagen RNeasy Mini Kit as per the manufacturer’s instructions, and reverse transcribed with iScript cDNA Synthesis Kit (Bio-Rad Laboratories, Hercules, CA). Quantitative RT-PCR was performed using Ssofast EvaGreen Supermix (Bio-Rad) with *Hprt1* as the reference gene, or TaqMan Fast Universal PCR Master Mix for *Ins1* and *Ins2* (Applied Biosystems) with mouse β-actin (4352341E, Thermo Fisher Scientific) as a reference gene, in a StepOnePlus real-time PCR system (Applied Biosystems, Foster City, CA). Transcript levels were quantified by the 2^−ΔΔCT^ method. All primer sequences are listed in Supplementary Table S2.

For miR-375 measurement in the circulation, extracellular RNA was isolated from plasma samples in the presence of an MS2 carrier RNA (Roche, Laval, Canada) using the Qiagen miRNeasy Kit. Reverse transcription was performed with the Universal cDNA Synthesis Kit II (Exiqon, Vedbaek, Denmark). Quantitative RT-PCR was performed using the ExILENT SYBR Green Master Mix (Exiqon) with LNA-based miRNA primers (Exiqon). Relative values were calculated with the ΔCT method using miR-16* as a reference miRNA.

### Assays

Insulin was measured in plasma, pancreas, and islets using the Mouse Ultrasensitive Insulin ELISA (ALPCO, Salem, NH). Insulin from perifusions was measured with the Rat Insulin RIA (Millipore, Billerica, MA). Proinsulin (Rat/Mouse Proinsulin ELISA, Mercodia AB, Uppsala, Sweden), leptin (Mouse Leptin ELISA Kit, Crystal Chem, Downers Grove, IL), and triglycerides (Serum Triglyceride Determination Kit, Sigma-Aldrich) were measured in plasma.

### Statistical analyses

Statistical analyses were performed using GraphPad Prism 7, with significance defined as p < 0.05. For the HFD study, all statistical comparisons were done within dietary condition. Area under the curve (AUC) measurements were conducted with the baseline set to a value of 0. Statistical tests are detailed in figure legends. T-tests were used for normally distributed data (two-tailed, unpaired unless otherwise stated in the figure legend). Bonferroni correction was used to correct for multiple comparisons, where applicable. Nonparametric tests (Mann-Whitney) were used for any data that did not pass the Shapiro-Wilk test for normality. Continuous data are represented by line graphs with median ± interquartile (IQR) range, and categorical data are represented by box and whisker plots. Where a panel contains a group with n = 3, floating bars represent the range of values and the line is at the median. For box and whisker plots and floating bar graphs, all individual data points are shown. For line graphs that show no indication of sample size such as a corresponding AUC graph, sample size is provided in the figure legend.

## Supplementary information

Supplementary information
