# Supplementary material for: Early overnutrition reduces Pdx1 expression and induces β cell failure in Swiss Webster mice
Source: Sci Rep. 2019 Mar 6;9:3619. doi: 10.1038/s41598-019-39177-3 (PMC6403421; doi:10.1038/s41598-019-39177-3)
Supplement: Supplementary file 1 — Supplementary information [file 41598_2019_39177_MOESM1_ESM.pdf]

Early overnutrition reduces Pdx1 expression and induces β cell failure in Swiss Webster mice

Maria M. Glavas, Queenie Hui, Eva Tudurí, Suheda Erener, Naomi L. Kasteel, James D. Johnson, Timothy J. Kieffer

Supplementary Information

Supplementary Table S1 Primary antibodies used

Protein / Peptide Target	Name of Antibody	Species Raised In; Monoclonal or Polyclonal	Manufacturer and/or Name of Individual Providing the Antibody	Catalog #	Dilution Used
Insulin	Guinea pig anti-insulin	Guinea pig; polyclonal	Millipore	4011-01F	1:1000
Insulin	Guinea Pig anti-insulin	Guinea pig; polyclonal	Sigma	I8510	1:1000
Insulin	Insulin (C27C9) rabbit mAb	Rabbit; monoclonal	Cell Signaling	3014	1:200
Glucagon	Glucagon antibody	Rabbit; polyclonal	Thermo Fisher Scientific	PA1-37768	1:100
Cleaved Caspase 3	Cleaved Caspase-3 (Asp175) antibody	Rabbit; polyclonal	Cell Signaling	9661	1:1000
Pdx1	Pdx1 antibody	Guinea pig; polyclonal	Abcam	ab47308	1:100
Aldh1a3	Rabbit anti-Aldh1a3	Rabbit; polyclonal	Novus Biologicals	NBP2-15339	1:100
L-myc	Rabbit anti-L-myc antibody	Rabbit; polyclonal	Abcam	Ab28739	1:200
CD3	Anti-CD3-epsilon antibody	Rabbit; polyclonal	AnaSpec	29588	1:100
CD45	CD45 antibody	Rat; monoclonal	BD Biosciences	550539	1:25

Supplementary Table S2. Primer sequences for quantitative RT-PCR

Gene	Primer Sequence (5’-3’)	
Ins1	Forward	GAAGTGGAGGACCCACAAGTG
	Reverse	ATCCACAATGCCACGCTTCT
	Probe	CCCGGGGCTTCCTCCCAGCT
Ins2	Forward	GAAGTGGAGGACCCACAAGTG
	Reverse	GATCTACAATGCCACGCTTCTG
	Probe	CCTGCTCCCGGGCCTCCA
Insr	Forward	AATGGCAACATCACACACTACC
	Reverse	CAGCCCTTTGAGACAATAATCC
Pdx1	Forward	TGGAAGAGCCCAACCGCGTCCA
	Reverse	CTCTCGGTCAAGTTCAACATCACTGCCAGCT
Foxo1	Forward	AGAAGAGGCTCACCCTGTCG
	Reverse	GCATCCACCAAGAACTCTTTCCAG
Neurod1	Forward	CTCCAGGGTTATGAGATCGTCACT
	Reverse	TTTCTTGTCTGCCTCGTGTT
Nkx6-1	Forward	CTTCGCCCTGGAGAAGAC
	Reverse	CCGAGTCCTGCTTCTTCTTG
Mafa	Forward	GGTGGAGGGTGTTATAAGCTGGGCGTATCT
	Reverse	CCTGTTCAGAAGACTCAGTGAGAGCCACCT
Srebp1c	Forward	ATCGGCGCGGAAGCTGTCGGGGTAGC
	Reverse	ACTGTCTTGGTTGTTGATGAGCTGGAG
Ucp2	Forward	TTCTGAGCCTCTCCATGCTG
	Reverse	GGGAGGTGAGGTGGGAAGTA
Plin2	Forward	ATTCTGAACCAGCCAACGTCCG
	Reverse	CTTATCCACCACCCCTGAGACTG
Chop	Forward	TATCTCATCCCCAGGAAACG
	Reverse	CTGCTCCTTCTCCTTCATGC
Nos2	Forward	CCAAGCCCTCACCTACTTCC
	Reverse	CTCTGAGGGCTGACACAAGG
Hprt1	Forward	GCTGACCTGCTGGATTACAT
	Reverse	TTGGGGCTGTACTGCTTAAC

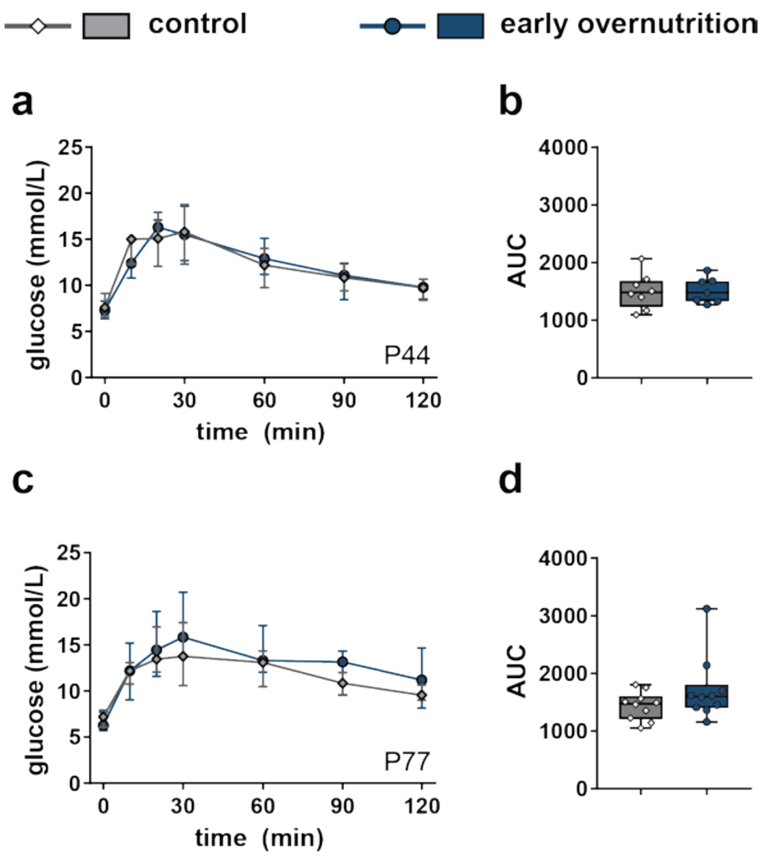

Supplementary Figure S1. Glucose tolerance tests in non-diabetic adults. Glucose tolerance tests were performed by oral gavage of 2 g/kg glucose in 6 hr fasted mice at postnatal day 44 (P44) (a), with corresponding AUC in (b), and at postnatal day 77 (P77) (c), with corresponding AUC in (d). Line graphs (a,c) represent median ± interquartile range and box and whisker plots (b,d) represent the interquartile range (box) and minimum to maximum values (whiskers), with line at the median. AUC: area under the curve with baseline = 0.
